# Immunomodulatory, Antioxidant, and Anti-Inflammatory Activities of Green Synthesized Copper Nanoparticles for Treatment of Chronic *Toxoplasma gondii* Infection

**DOI:** 10.3390/ph16111574

**Published:** 2023-11-07

**Authors:** Abdullah D. Alanazi, Sultan F. Alnomasy

**Affiliations:** 1 Department of Biological Sciences, Faculty of Science and Humanities, Shaqra University, P.O. Box 1040, Ad-Dawadimi 11911, Saudi Arabia; 2 Department of Medical Laboratories Sciences, College of Applied Medical Sciences, Shaqra University, Al-Quwayiyah 19257, Saudi Arabia; s.alnomasy@su.edu.sa

**Keywords:** nanoparticles, toxoplasmosis, in vitro, in vivo, inflammation, cytokines

## Abstract

Background: Nowadays, interest in the use of nanotechnology for medical purposes is increasing. The current experimental investigation is planned for the green synthesis, characterization, and efficacy of copper nanoparticles (CLN) against chronic *Toxoplasma gondii* infection. Methods: Green synthesis of CNP was performed using the *Lupinus arcticus* extract via the precipitation method. The effects of CNP on tachyzoites, infectivity rate, parasites inside THP-1 cells, nitric oxide (NO) triggering, iNOS, and IFN-γ expression genes were evaluated. Following toxoplasmosis in BALB/c mice via the *T. gondii* ME49 strain, mice received CNP at 5 and 10 mg/kg/day alone and combined with pyrimethamine (PYM) at 5 mg/kg for two weeks. CNP’s in vivo effects were evaluated by analyzing the load and size of cysts, oxidant/antioxidant enzymes, and bradyzoite surface antigen 1 (BAG1) expression gene levels. Results: CNP displayed a circular shape ranging from 10 to 85 nm. The IC50 value of CNP and PYM against tachyzoites was 37.2 and 25.7 µg/mL, respectively, whereas the CC50 value of CNP and pyrimethamine against THP-1 cells was 491.4 μg/mL and 269.5 μg/mL, respectively. The rate of infectivity and parasite load among THP-1 cells exposed to CNP was obviously reduced (*p* < 0.05). CNP at the doses of 5 and 10 mg/kg predominantly along with PYM evidently (*p* < 0.05) reduced the number and size of the *T. gondii* cysts in the infected mice. The levels of NO, iNOS, and IFN-γ genes were remarkably (*p* < 0.001) boosted compared with the cells without treatment. CNP at the doses of 10 and 20 mg/kg drastically (*p* < 0.05) reduced the oxidative stress markers in the infected mice, whereas CNP significantly elevated the level of antioxidant factors. CNP also revealed no toxicity in the liver and kidney at the tested doses in healthy mice. Conclusions: Our experimental study reported the beneficial effects of CNP principally along with existing chemical drugs against latent toxoplasmosis in mice, whereas the possible action mechanisms of CNP are controlling oxidative stress, refining antioxidant enzymes, and increasing the production of immunomodulatory cytokines with no toxicity to the function of vital organs. But, additional trials are required to confirm these results, as well as to clarify the accurate mechanisms and their toxicity.

## 1. Introduction

*Toxoplasma gondii* infection (TGI) is recognized as a highly prevalent parasitic disease in humans and other warm-blooded animals, especially in tropical and subtropical regions [1,2]. Humans are infected by eating cysts from raw meat (e.g., beef and pork), ingesting oocysts in food contaminated with cat feces, and placental diffusion [3]. TGI may be observed as acute or chronic, with or without signs; however, with the immune system activation, the parasite stays put in tissue cyst forms, gently reproduces host organs, and initiates a latent TGI [4]. Therefore, in immunocompetent persons, TGI is routinely asymptomatic, whereas it is deadly in patients with immune system defects due to reactivation of the infection and subsequent lethal symptoms, e.g., encephalitis [5]. Pyrimethamine (PYM), mainly in combination with sulfadiazine, is still reported as the most effective chemical agent against TGI [6]. This drug is an antagonist of folic acid and can result in the dose-linked suppression of the bone marrow, which is mitigated by parallel administration of folic acid [6]. In addition, there are serious challenges in TGI chemotherapy, such as the inability of common drugs to control and eliminate parasites inside the cyst and eradicate the infection, and the occurrence of adverse side effects, such as osteoporosis, blood poisoning, teratogenicity, and hematuria [7,8,9,10]. Accordingly, it is required to improve the current agents as well as find new agents for the prevention and control of TGI.

Nowadays, interest in using nanotechnology for medical purposes is increasing. Due to having exceptional possessions, such as small size and surface reaction, nanoparticles can be considered as potent candidates in the treatment of numerous diseases, mainly infectious ones [11,12]. Among the metal nanoparticles, copper nanoparticles (CNP) have drawn a lot of interest because of having various biological properties, e.g., antimicrobial, anti-inflammatory, anticancer, and antioxidant [13]. There are different methods for making nanoparticles of various materials with the possibility of controlling their size, composition, and uniformity [14]. The usage of herbal extracts in the phytosynthesis of nanoparticles, which is known as green synthesis, can be used as a nature-friendly method and a suitable alternative to conventional methods such as physical and chemical methods [15]. *Lupinus arcticus* L. is a plant that belongs to the Fabaceae family and displays various pharmacological effects, such as antidiabetic, anticancer, insecticide, and antifungal effects [16,17]. Despite conducting various studies on the anti-*Toxoplasma* effects of nanoparticles, different results have been obtained, which is probably due to the synthesis approaches, the parasite strain, and the way they are used [18,19]. The current experimental investigation was planned for the green synthesis, characterization, and efficacy of CNP against *T. gondii* infection.

## 2. Results and Discussion

**Figure 1:**
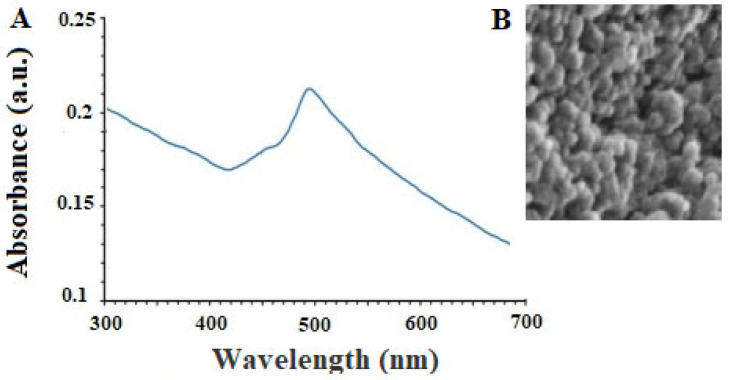
The results of the analysis of UV-Vis (**A**) and scanning electron microscope (**B**) of the obtained copper nanoparticles.

The peak of absorption of CNP acquired from the UV-Vis was observed at 491 nm (Figure 1A). UV-Vis spectroscopy is often used to determine the surface plasmon resonance (SPR) of nanoparticles, which is sensitive to electron oscillations with increased nanoparticle intensity. Due to the increase in surface electrons, the SPR wavelength changes with light absorption in the UV-vis range in metal nanoparticles. Therefore, the appearance of distinct peaks in the known region indicates the formation of nanoparticles. Here, a single peak at 491 nm can be attributed to CNP in the sample solution [20,21].

SEM analysis also showed that the green synthesized CNP displayed a circular shape with a size ranging from 10 to 85 nm, whereas, the largest spreading of particle size was observed at 30–50 nm (Figure 1B). SEM imaging can be used to determine the nanoparticle morphology and size. Consequently, these two variables are affected by construction conditions, so size and shape can be altered by modifying any environmental factors. Nanoparticles’ stability and biological properties are proportional to their size. Due to their greater surface-to-volume ratio, nanoparticles with a smaller size are more stable and aggregate less frequently. In contrast, coating nanoparticles with molecules can improve their biocompatibility and biological activity. The therapeutic activity of nanoparticles is substantially increased by reducing their size to below 100 nm [22]. This research produced copper nanoparticles smaller than 100 nm, which are suitable for antiparasitic applications [22].

**Figure 2:**
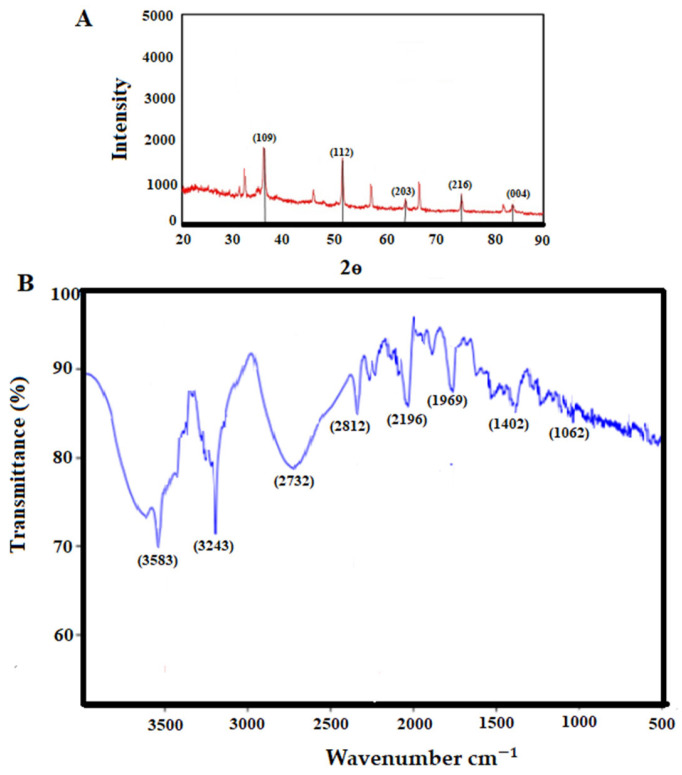
The analysis of X-ray diffraction (**A**) and Fourier transform infrared spectroscopy of the obtained copper nanoparticles (**B**).

The findings of XRD analysis showed the presence of a diffraction peak at 38.3°, 51.2°, 62.2°, 73.1°, and 83.3° corresponding to (109), (112), (203), (216), and (004), respectively, which represented the monoclinic crystalline phase of CNP (Figure 2A). X-ray diffraction provides data regarding the crystalline phases of the nanoparticles, thereby revealing the nanoparticles’ homogeneity, purity, and crystallite size. In this investigation, the XRD pattern provided specific signals that implied that the purity of the nanoparticles was satisfactory [23].

FTIR spectroscopy is used to assess the function of reactants in the production of nanoparticles. In fact, the FTIR spectrum reveals the functional groups of the coating and stabilizing chemicals involved in nanoparticle creation. These compounds significantly improve the biological performance of nanoparticles [24]. The bands at 3583, 3243, 2312, 2196, 1769, 1402, and 1062 cm^−1^ are related to the reaction of the plant extract with copper ions, O-H stretching of alcohol and phenol, C-H stretching of the aliphatic group, C=O stretching of ester carbonyl, C=C stretching of the aromatic ring, and C-O stretching of ester, respectively (Figure 2B). This demonstrates that the biomolecules of the extract play a role both as a reducer and as a coating for copper nanoparticles, so they can protect the CNP against oxidation and transformation into copper oxide.

**Figure 3:**
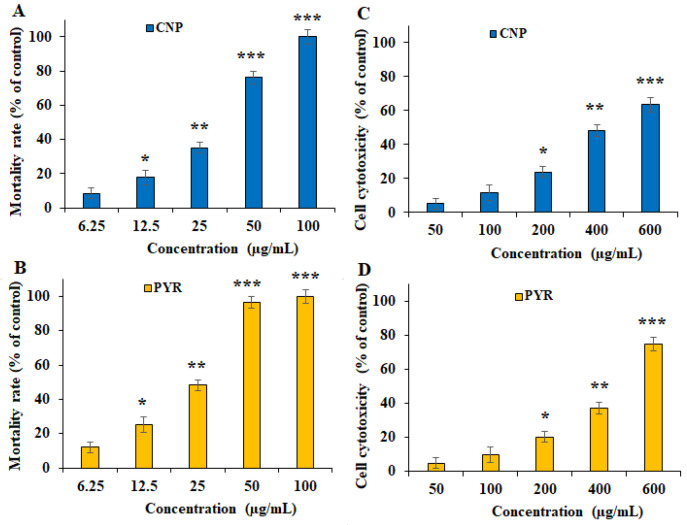
In vitro inhibitory effects of green synthesized copper (CNP) against *Toxoplasma gondii* tachyzoites (**A**) and THP-1 cells (**C**) compared to the pyrimethamine against *T. gondii* tachyzoites (**B**) and THP-1 cells (**D**). * *p* < 0.01, ** *p* < 0.01, and *** *p* < 0.001 compared to the control.

**Figure 4:**
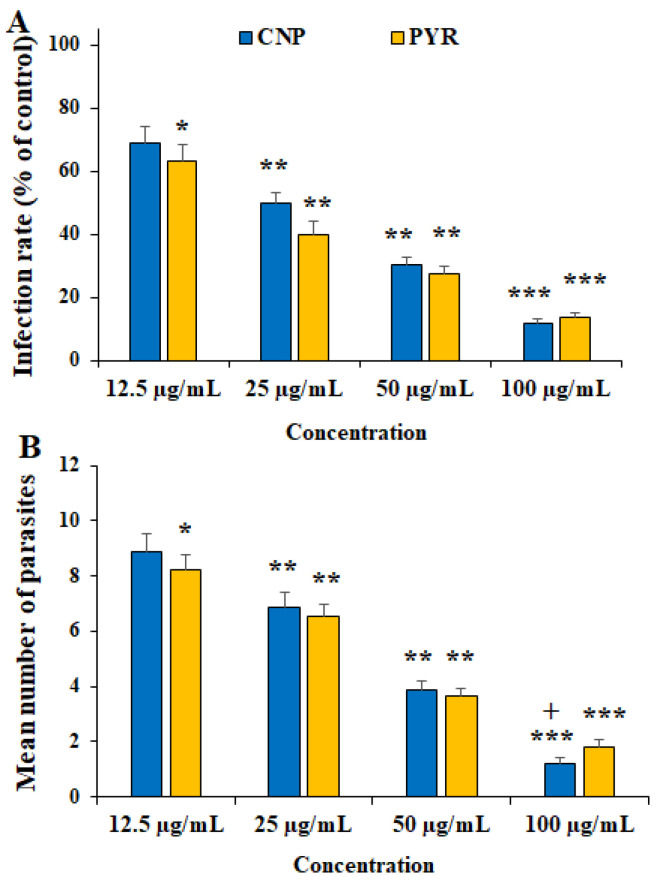
Effect of copper nanoparticles (CNP) and pyrimethamine (PYM) on the infectivity rate (**A**) and parasites inside THP-1 cells (**B**). * *p* < 0.05, ** *p* < 0.01, and *** *p* < 0.001 compared to the control (normal saline). + *p* < 0.05 compared to the PYM.

Figure 3 and Figure 4 show the inhibitory effects of CNP on *T. gondii* tachyzoite forms and THP-1 cells. CNP dose and time dependence diminished the viability of tachyzoites and THP-1 cells compared to normal saline. The IC_50_ value of CNP and PYM against tachyzoites was 37.2 and 25.7 µg/mL, respectively. The CC_50_ value of CNP and PYM on THP-1 cells was 491.4 μg/mL and 269.5 μg/mL, respectively. Following the exposure of THP-1 cells to CNP at 12.5, 25, 50, and 100 µg/mL, the rate of infectivity was evidently (*p* < 0.05) reduced by 68.8, 49.8, 30.2, and 11.9%, respectively; this exposure significantly reduced the number of intracellular parasites in THP-1 cells. In a study, Malekifard et al. (2020) showed the potent in vitro inhibitory effects of CNP 0.6 mg/mL against *Giardia deodenalis* cyst [25]. Albalawi et al. (2021) also reported that green synthesized CuNPs significantly inhibited the growth rate of *Leishmania major* amastigotes in a dose-dependent manner with an IC_50_ value of 116.8 μg/mL [26]. Another study conducted by Saad et al. (2015) demonstrated that CNP significantly reduced the viability of *Entamoeba histolytica* cysts and *Cryptosporidium parvum* oocysts with LC_50_-3 h of 0.13 and 0.72 mg/l, respectively [27]. These variations in the obtained results are associated with the type of tested parasites, the synthesis method of nanoparticles, and the type of assessment test [26]. Considering the antimicrobial mechanisms of action of CNP, previous studies reported that these nanoparticles displayed antimicrobial effects by provoking reactive oxygen species, disrupting cell walls, increasing cell membrane permeability, disrupting protein and DNA synthesis, restricting cell division by hindering the creation of the cell-septum-forming ring, as well as provoking the caspases activity and subsequently triggering programmed cell death [27]. The reported antimicrobial effect of CNP is not merely due to their release of metal ions but can also be attributed to their morphology, mainly their small size and high surface area to volume ratio, which allows them to interact closely with the microbial membranes of each bacterium [28].

**Figure 5:**
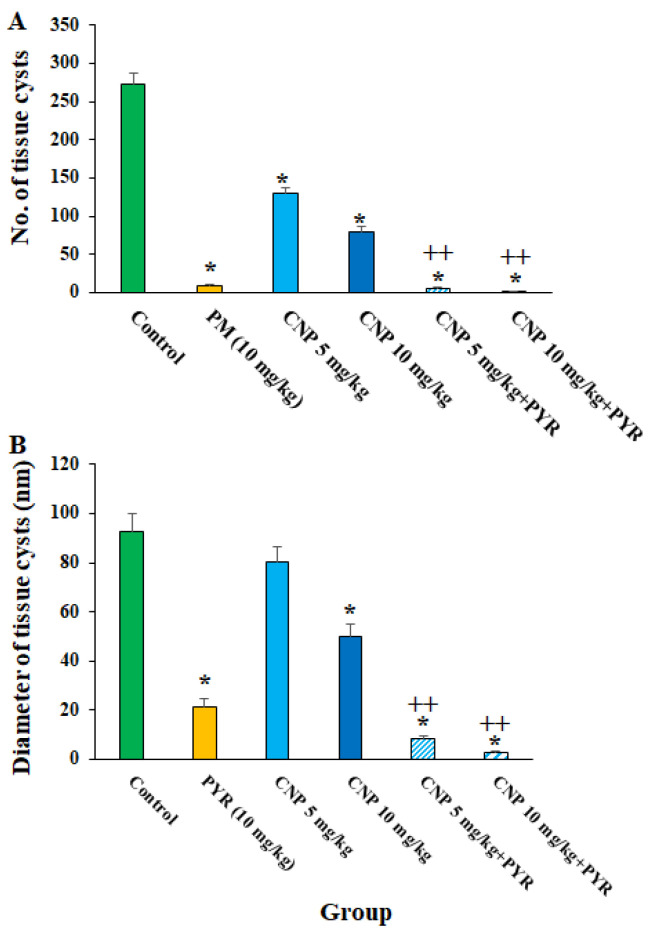
The number (**A**) and size (**B**) of the *T. gondii* cysts after treatment with copper nanoparticles (CNP) at the doses of 5 and 10 mg/kg alone in combination with pyrimethamine (5 mg/kg) for two weeks. * *p* < 0.001 compared to the control (normal saline). ++ *p* < 0.01 compared to the PYM.

Figure 5 shows the effects of CLN alone and in combination with PYM on the number and size of the tissue cysts in *T. gondii*-infected mice. CNP at the doses of 5 and 10 mg/kg principally along with PYM noticeably (*p* < 0.05) reduced the number and size of the *T. gondii* cysts in the infected mice. With respect to the effects of green synthesized CNP against pathogenic parasites, Ezzatkhah et al. (2021) showed that green synthesized CNP using *Capparis spinosa* extract at 75 mg/mL along with albendazole killed the *Echinococcus granulosus* protoscoleces by provoking the caspase activity of this parasite [29]. In addition, Albalawi et al. revealed that green synthesized CNP alone and along with glucantime obviously inhibited and controlled the replication of *Leishmania major* promastigote and amastigote stages, whereas predominantly recovered the cutaneous *Leishmania* lesions in BALB/c mice [26]. Consistent with the study conducted by Albalawi et al. (2021), the treatment of mice infected with *T. gondii* Tehran strain by green synthesized CNP at doses of 2 and 4 mg/kg for two weeks markedly controlled the T. gondii infection in mice by reducing the number and size of tissue cysts [29]. Our results, in parallel with other antiparasitic studies of CNP, showed that CNP had potent in vivo antiparasitic efficacy against some pathogenic parasite strains; however, these differences in the obtained results are due to some factors, such as the type of parasite, the synthesis method of nanoparticles, and the doses used [26,30].

**Figure 6:**
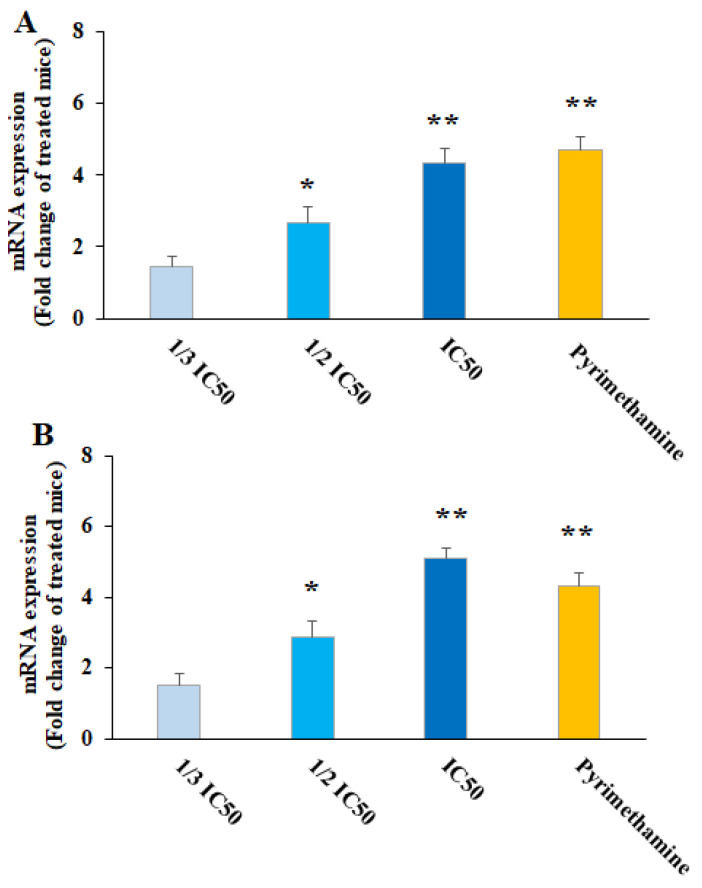
Effect of copper nanoparticles (CNP) and pyrimethamine (PYM) on iNOS (**A**) and IFN-γ (**B**) gene expression in THP-A cells. * *p* < 0.01 and ** *p* < 0.001 compared with control normal saline group.

Nitric oxide is the main metabolite released by various immune cells after being triggered by cytokines, which play a key function in the protection against several parasites, especially intracellular ones [31]. Accordingly, the production of cell-mediated immunity and NO triggering is well-known as a promising strategy for the design and discovery of new agents for the prevention and treatment of parasitic infections [31]. NO was produced by IFN-γ, a crucial cytokine that controls toxoplasmosis both in vitro and in vivo [32]. Our results showed that after the treatment of THP-1 cells with CNP at 1/3 IC50, ½ IC50, and IC_50_, the NO level was 4.13 ± 0.33, 8.5 ± 1.15, and 12.3 ± 1.55 nM (*p* < 0.05), respectively; this value for the cells treated with normal saline and IFN-γ + LPS was 3.24 ± 0.16 and 29.7 ± 4.15 nM, respectively. As depicted in Figure 6, following the treatment of *T. gondii*-infected mice with CNP, a considerable upregulation of iNOS and IFN-γ genes was observed (*p* < 0.001), predominantly at ½ IC50 and IC50, compared to the mice treated with normal saline (Figure 6). Consistent with our results, Albalawi et al. (2021) showed that the treatment of mice infected with *T. gondii* Tehran strain by green synthesized CNP at doses of 2 and 4 mg/kg for two weeks significantly controlled the TGI by increasing the expression of cellular immunity cytokines IFN-γ, IL-12, and iNO [26]. Currently, targeted host-directed immunotherapy aimed at activating or suppressing specific elements of the immune system is considered for the treatment of toxoplasmosis [33]. Considering the effect of CNP on strengthening the immune system, previous studies showed that CNP displayed their strengthening effect on the immune system via the maturation of dendritic cells and by provoking the release of cytokines IL-12 and T-cell derived cytokines IFN-γ, IL-4, IL-6, TNF-α, and IL-1β [34]. This suggests that CNP can control and prevent *T. gondii* infection in mice by activating cellular-mediated immunity (the central immunity factor against *T. gondii*).

**Figure 7:**
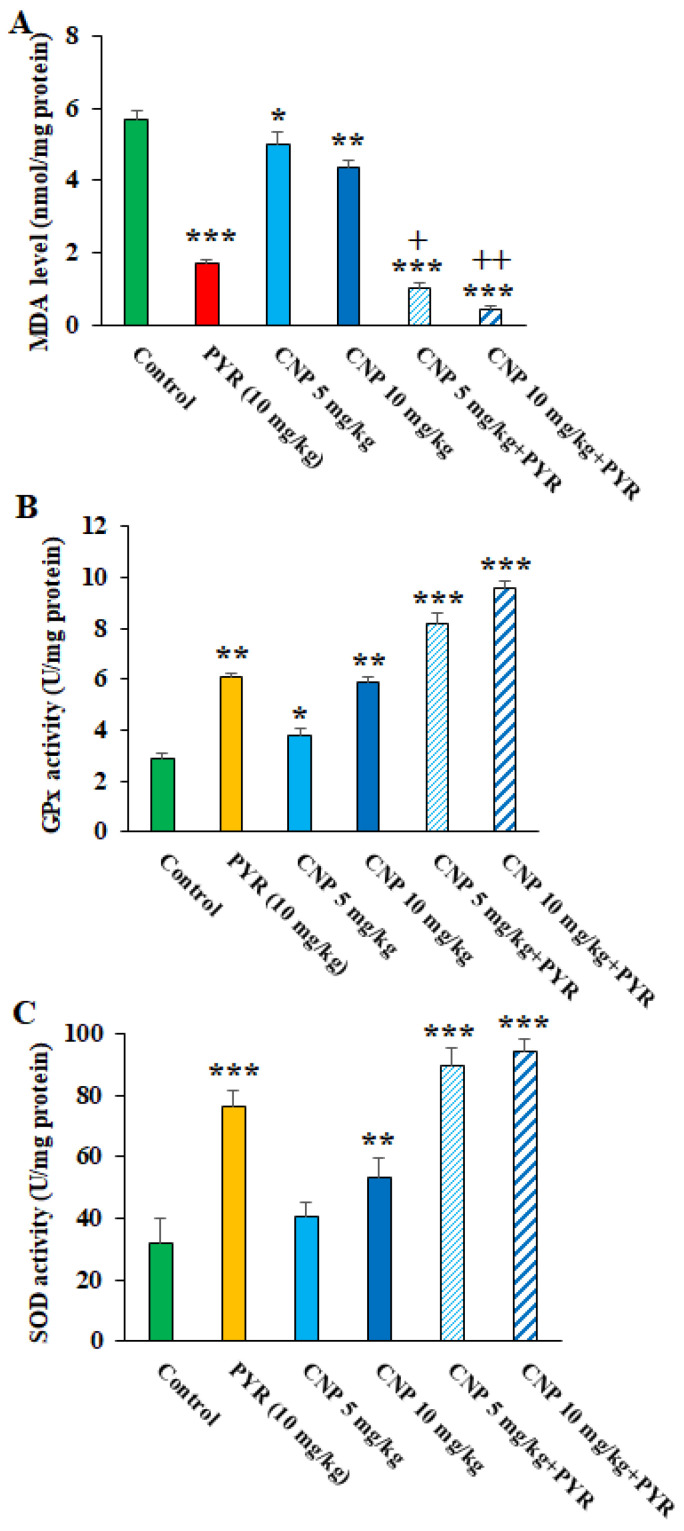
Effects of copper nanoparticles (CNP) treatment on the malondialdehyde (**A**), glutathione peroxidase (**B**), and superoxide dismutase enzyme activity (**C**) in infected mice. * *p* < 0.05, ** *p* < 0.01, and *** *p* < 0.001 compared to the control (normal saline). + *p* < 0.05 and ++ *p* < 0.01 compared to the pyrimethamine (PYM).

Oxidative stress (OS) plays a crucial role in the pathogenesis of TGI in the host [35]. Once TGI starts, tissue damage occurs by provoking LPO production and accordingly spreading the free radicals [36]. We found that CNP at 5 and 10 mg/kg, mostly along with PYM, apparently (*p* < 0.05) diminished the oxidative stress in the liver of TGI mice; however, these treatments caused a significant elevation in the antioxidant factors of SOD and GPx (Figure 7), thereby demonstrating that CNP controlled the TGI in mice by decreasing the OS and increasing the antioxidant system. Similarly, Tavakoli et al. (2023) demonstrated the promising anti-inflammatory and antioxidant effects of green synthesized CNP by *Artemisia annua* extract in mice with second-degree burns by increasing the activity of SOD, CAT, and GPX enzymes as well as significantly reducing the MDA level [37]. It has been proven that the enzymatic antioxidant defense is one of the mechanisms that protect the host cells against an excess of free radicals due to parasitic infections such as *T. gondii* [38]. Since the antioxidant effects of CNP have been proven to control oxidative stress both in the present study and in previous studies, it can be suggested that these nanoparticles are able to control *T. gondii* infection in mice by inhibiting oxidative stress.

**Figure 8:**
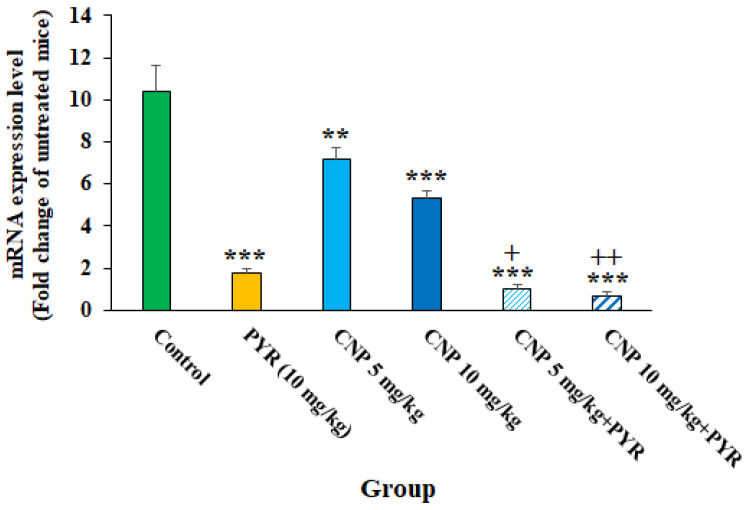
The effects of copper nanoparticles (CNP) alone and in combination with pyrimethamine (PYM) on the expression of the BAG1 gene in infected mice; ** *p* < 0.01 and *** *p* < 0.001 compared to the control (normal saline); + *p* < 0.05 compared to PYM; ++ *p* < 0.01 compared to PYM.

Based on the molecular tests, although TGI resulted in an increase in the expression of the BAG1 gene, following the treatment of TGI mice with CNP at 5 and 10 mg/kg alone and along with PYM, the expression of the BAG1 gene (*p* < 0.05) was evidently decreased (Figure 8). Previously, it was shown that disorder and reduction in the *T. gondii* bradyzoite-specific gene BAG1 results in significant reductions in in vivo cyst formation and bradyzoite differentiation [36]. Therefore, it can be proposed that CNP is probably able to control *Toxoplasma* infection in mice by reducing BAG1 gene expression and subsequently reducing the formation of tissue cysts.

**Figure 9:**
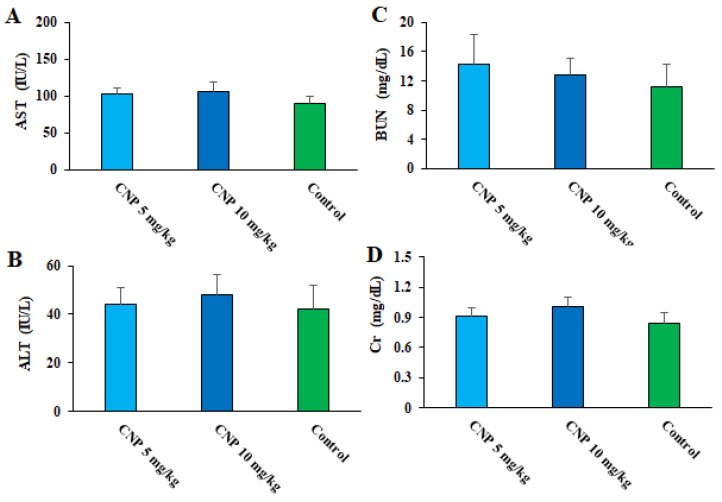
Effects of copper nanoparticles (CNP) on liver functional factors of aspartate transaminase (**A**) and alanine transaminase (**B**), as well as some kidney functional factors such as blood urea nitrogen (**C**) and creatinine (**D**) in healthy mice.

Currently, it has been proven that some factors such as the different methods of synthesis, which lead to the size, shape, and other different physical and chemical properties of the NP, can affect the toxicity of the synthesized nanoparticles [39,40]. Therefore, toxicity and optimal doses for administration should be evaluated in animal models. Regarding the toxicity effects of CNP on the markers of liver and kidney function in healthy mice, the biochemical tests demonstrated that although in some cases an increase was observed, these differences were not significant compared to the mice that received normal saline (Figure 9). Similarly, Sulaiman et al. (2018) showed that the oral administration of green synthesized Cu NPs to male Swiss albino mice had no significant toxicity in the liver, kidney, spleen, and body weight up to 400 mg/kg [41]. Accordingly, we can suggest that the oral administration of these green synthesized nanoparticles at doses of 5 and 10 mg/kg for 14 days had no toxicity on the function liver and kidney of the tested mice.

## 3. Materials and Methods

### 3.1. Green Synthesis of CNP

#### 3.1.1. Plant Extract

*Lupinus arcticus* materials (aerial parts) were prepared in May 2023 from a plant shop in Riyadh, Saudi Arabia. The plant was identified and saved as the voucher specimen (No. 52-2022) at Shaqra University, Saudi Arabia. For extraction, 250 g of the material was extracted using water for 72 h at 21 °C via a percolation procedure [42,43].

#### 3.1.2. Synthesis of CNP

Based on the precipitation technique, after adding the aqueous extract (50 mL), a beaker containing copper sulfate solution (100 mL, 1 mM) was mixed for 10 min, and the combination was kept at 24 °C after 12 h. The color alteration of the mixture to dark yellow with the creation of turbidity resulted in CNP synthesis [44].

#### 3.1.3. UV-Vis Spectroscopy Analysis

To investigate the alteration of the Cu ions to CNP, the NPs solution (300 µL) was diluted with normal saline (3 mL) and was studied using UV–vis spectrum using a spectrophotometer tool (Shimadzu UV2550, Nagoya, Japan, 300–700 nm).

#### 3.1.4. Physical Characterization of CNP

The size and shape of the green synthesized CNP were assessed using a scanning electron microscope (SEM, Mira3, Tescan, Brno, Czech Republic—15 kV, amplification of 10×, and 1 nm) and a Dynamic light scattering (DLS) device (Zetasizer, Malvern Instruments Ltd., Malvern, UK).

#### 3.1.5. X-ray Diffraction (XRD) Study

The presence of copper in green synthesized CNP and its crystal organization was assessed using an XRD apparatus (APD 2000, G.N.R. S.r.l., Agrate Conturbia, Italy) with a copper lamp (Ka-ray source) at a wavelength of λ = 1.54 A0.

#### 3.1.6. Fourier Transform Infrared Spectroscopy (FTIR) Analysis

The FTIR analysis was performed to identify the biomolecules responsible as coating agents for the synthesis of nanoparticles. In brief, the CNP powder was mixed with potassium bromide to yield tablets, which were studied using a device (Tensor 27, Bruker, Karlsruhe, Germany).

### 3.2. Parasite

*T. gondii* (ME49) was obtained from Shaqra University, Saudi Arabia, and was passaged and kept via intraperitoneal infusion in BALB/c mice.

### 3.3. Cell Culture

The human macrophage cell lines (THP-1) were cultured in a RPMI-1640 medium enriched with fetal bovine serum (10%) and pen/strep antibiotics (100 units/mL) at 37 °C with 5% CO_2_.

### 3.4. In Vitro Anti-Toxoplasma Effects of CNP

#### 3.4.1. Effects of CNP on *T. gondii* Tachyzoite Forms

Cell inhibitory MTT test was utilized to investigate the effects of CNP on tachyzoites of *T. gondii* (21). In summary, tachyzoites (1,000,000/mL) were treated with CNP (12.5 to 100 µg/mL) in 96-well plate for 72 h at 37 °C. Then, the MTT (Sigma-Aldrich, Hamburg, Germany) solution (5 mg/mL) was mixed, and the plates were kept again under the same conditions. Next, dimethyl sulfoxide was added as the stop solution, and the optical density (OD) of the combinations was recorded at 570 nm utilizing an ELISA reader (LX800; Biotec, Winooski, VT, USA). A solvent (normal saline) and pyrimethamine (PYM) were utilized as control groups.

#### 3.4.2. Cytotoxicity Effects of CNP on THP-1 Cells

The cytotoxicity activity of CNP on THP-1 cells was determined by assessing the 50% cytotoxic concentration (CC50) using an MTT assay based on the method described in the previous section [45,46].

#### 3.4.3. Effect of CNP on Infectivity Rate of THP-1 Cells

At first, tachyzoites (1,000,000 cells/mL) were treated with CNP at 12.5–100 µg/mL at 24 °C for 240 min. Subsequently, THP-1 cells (100,000/mL) were subjected to treated parasites at 37 °C for 24 h. When preparing the slides and staining them with Giemsa, the percentage of infected cells was examined under a light microscope [47].

#### 3.4.4. Effect of CNP on Parasites Inside THP-1 Cells

The THP-1 cells (100,000/mL) were seeded in a 24-well plate and preserved for 24 h at 37 °C. Then, the cells were subjected to tachyzoites (1,000,000/mL) at a proportion of 1 to 10 for 24 h. Next, the infected cells were treated with CNP at 12.5–100 µg/mL for 4 h. Once the slides were prepared and stained with Giemsa, the number of parasites was examined under a light microscope [47]. Normal saline and PYM were utilized as control groups.

#### 3.4.5. Estimating the Nitric Oxide (NO) Generating

The THP-1 cells (100,000/mL) were treated with CNP for 48 h. Next, 0.1 mL of the superior part of the mixture was purred to the 0.1 mL of Griess reagent (Sigma-Aldrich, Germany) in a 96-well plate. The OD of the mixture was studied at 540 nm using an ELISA reader [29]. The solvent (normal saline and lipopolysaccharide (10 ng/mL) + IFN-γ (10 U/mL) were utilized as the negative and positive controls, respectively.

#### 3.4.6. Assessing the Activity of CNP on iNOS and IFN-γ Expression Genes

The THP-1 cells (100,000/mL) were treated with CNP for 48 h. Total RNA was isolated using the commercial kit procedures (Qiagen, Hilden, Germany). The obtained RNAs were transcribed utilizing the Fermentas kit (Thermo Fisher Scientific, Waltham, MA, USA). Next, the products were rated using SYBR green real-time PCR based on the primers described elsewhere [48]. Basic denaturation was defined at 95 °C for 10 min, followed by 40 extension cycles and a separate cycle at 72 °C for 5 min. Finally, 2^ΔΔCt−^ was estimated utilizing Bio-Rad iQ5 Optical System Software, version 2.1 (Hercules, CA, USA).

### 3.5. In Vivo Anti-Toxoplasma Effects

#### 3.5.1. Animals

In total, 102 male BALB/c mice were acquired from Shaqra University, Saudi Arabia. The mice were maintained at a standard temperature and humidity with satisfactory water and food ad libitum.

#### 3.5.2. Ethics

This investigation was designed and approved by the ethical committee of Almaarefa University, Saudi Arabia (Number. IRB07-18052022-64).

#### 3.5.3. *T. gondii* Infection Induction in Mice and Their Treatment

To induce *T. gondii* infection in mice, 0.5 mL of a brain suspension with 20–25 cysts and pen/strep antibiotics was intraperitoneally injected into mice [49]. One day post-*T. gondii* infection induction, 72 infected mice were assigned to six groups, covering 12 mice that received the drugs for 14 consecutive days, including the following:

- Normal saline.
- PYM at 10 mg/kg (PYM).
- CNP at 5 mg/kg.
- CNP at 10 mg/kg.
- CNP at 5 mg/kg + PYM
- CNP at 10 mg/kg + PYM

#### 3.5.4. Assessing the Efficacy of CNP Therapy on Oxidant/Antioxidant Enzymes

One day after the treatment, half of the mice in each group (six mice) were euthanized. After collecting the liver tissue from each mouse, the liver homogenates were used to determine the oxidant/antioxidant enzymes. The hepatic level of lipid peroxidation (LPO), glutathione peroxidase (GPx), and superoxide dismutase enzyme activity (SOD) as the oxidant/antioxidant enzymes were examined according to the Abcam kits (Waltham, MA, USA).

#### 3.5.5. Brain Tissue Collection

Eight weeks post-infection, the remaining mice from each group (six mice) were anesthetized via (i.p. infusion of ketamine+ xylazine (15 and 100 mg/kg), and then the entire brain tissue was collected.

#### 3.5.6. Effect of CNP Treatment on Parasite Load

Initially, smears were obtained from the right brain hemisphere, and then the number and dimensions of *T. gondii* tissue cysts were recorded utilizing light microscopy [48].

#### 3.5.7. Effects of CNP Treatment on the Bradyzoite Surface Antigen 1 (BAG1) Gene

First, the total mRNA was isolated from the left-brain hemisphere of the mice using the Qiagen commercial mRNA extraction kit (Germany). The obtained RNAs were transcribed utilizing the Fermentas kit, USA. Next, the amplification products were rated using SYBR green real-time PCR based on the primers described elsewhere. Basic denaturation was defined at 95 °C for 10 min, followed by 40 extension cycles and a separate cycle at 74 °C for 4 min. Finally, 2^ΔΔCt−^ was estimated utilizing Bio-Rad iQ5 Optical System Software, USA [48].

### 3.6. Safety of the Green Synthesized CNP

Thirty healthy mice were allocated into three groups (with 10 mice per group): (i) receiving normal saline for 14 days; (ii) receiving CNP daily at 5 mg/kg for 14 days; and (iii) receiving CNP daily at 10 mg/kg for 14 days. Twenty-four hours post-treatment, the animals were anesthetized utilizing a combination of ketamine and xylazine at a ratio of 100:10 mg/kg. After collecting the blood samples from the hearts of the tested mice and obtaining the serum of specimens, they were tested using commercial kits of Roche, Germany, to diagnose kidney factors of creatinine and blood urea nitrogen as well as liver function markers, such as ALT and AST [50].

### 3.7. Statistical Analysis

All trials were performed in triplicate to increase the reliability of the results. The acquired data were analyzed using SPSS software version 26.0, whereas ANOVA and a t-test were applied to measure the investigated groups. *p* < 0.05 was finally measured as a significant difference.

## 4. Conclusions

Our experimental study reported the beneficial effects of CNP principally along with existing chemical drugs against latent TGI in mice, whereas the possible action mechanisms of CNP include controlling oxidative stress, refining antioxidant enzymes, and increasing the production of pro-inflammatory cytokines with no toxicity to the function of vital organs. Nonetheless, additional trials to confirm these results, as well as to clarify the accurate mechanisms and their toxicity, are mandatory.

## Data Availability

All data generated or analyzed during this study are included in this published article.
